# Accounting the Role of Prosociality in the Disjunction Effect with a Drift Diffusion Model

**DOI:** 10.3390/bs16010132

**Published:** 2026-01-16

**Authors:** Xiaoyang Xin, Bo Liu, Bihua Yan, Ying Li

**Affiliations:** 1 Preschool College, Luoyang Normal University, Luoyang 471934, China; 2 Center for Psychological Sciences, Zhejiang University, Hangzhou 310012, China; 3 Institute of Psychology and Behavior, Henan University, Kaifeng 475001, China; liubo2023@henu.edu.cn; 4 School of Psychology, Shaanxi Normal University, Xi’an 710062, China; yanbihua@snnu.edu.cn (B.Y.); liying@snnu.edu.cn (Y.L.)

**Keywords:** disjunction effect, prisoner’s dilemma game, prosociality, social value orientation, drift diffusion model

## Abstract

The disjunction effect in the prisoner’s dilemma game shows that humans tend to cooperate more under uncertain condition (U) than under the two complementary known conditions—one being competitive (D) and the other being cooperative (C)—a well-known violation of the classical decision principle. Our study explores the potential role of prosociality in the disjunction effect. We measured prosocial trait via the SVO Slider Measure, and prosocial bias via the drift diffusion model (DDM). By using the SVO Slider Measure (for prosocial trait) and the DDM starting-point bias parameter (for prosocial bias), we found that the variation in prosocial bias between uncertain and certain conditions substantially contributes to the disjunction effect. At the aggregate level, prosocial bias significantly decreased from U to D (competitive) but did not differ between U and C (cooperative). At the individual level, participants showed heterogeneous bias changes across prosocial-trait groups: intermediate participants had the largest bias shifts. This heterogeneity underlies the observed inverted U-shaped relationship between prosocial trait and effect size of the disjunction effect. Our study fills a critical gap by clarifying how prosocial inclination influences the disjunction effect.

## 1. Introduction

It is interesting that human behavior does not always square with theoretical predictions, especially for the circumstances under uncertainty. One well-known example is the disjunction effect in the prisoner’s dilemma (PD) game that was first discovered by Shafir and Tversky (1992). In that work, participants cooperated more under the uncertain condition (U, where they did not know their opponent’s action) than under the two known conditions (D and C, where they were told their opponent would defect or cooperate, respectively). This violates the principle that the cooperation rate under U should lie between those in the known conditions (Savage, 1954).

Since the discovery of the disjunction effect in the Prisoner’s Dilemma, the phenomenon has attracted substantial attention and has been examined extensively (Shafir & Tversky, 1992; Croson, 1999; Li & Taplin, 2002; Li et al., 2007; Hristova & Grinberg, 2008; Hristova & Grinberg, 2010). Shafir and Tversky (1992) proposed the “reluctance-to-think” explanation for the disjunction effect in the PD game. They suggested that under condition U, participants cannot fully consider all of the relevant branches of a decision tree, therefore sometimes fail to discover their optimal choice (defection). Li and Taplin (2002) and Li et al. (2007) proposed “equate-to-differentiate” theory to explain the disjunction effect, stating that under condition U, participants tend to consider less about the differences between their own payoffs but more about the differences between opponents’ payoffs when making their choices, leading to the preference for the option (cooperation) that maximizing opponents’ payoff. Those above-mentioned explanations, though diverse, commonly attribute the disjunction effect to changes in the deliberative preference strength toward defection when moving from certain to uncertain informational contexts.

Despite the progress made in previous studies, prosociality (including prosocial trait and prosocial bias), has been largely overlooked as an important factor in social economic decision-making. Prosocial trait refers to relatively stable personality characteristics that do not vary across conditions. An individual with higher prosocial trait is more likely to act cooperatively in social decision-making (Balliet et al., 2009; Thielmann et al., 2020). Prosocial bias, in contrast, reflects an intuitive tendency toward prosocial action that directly influences the decision process and can shift across situational contexts (Zaki & Mitchell, 2013; Bear & Rand, 2016), which can vary across different conditions (e.g., time-free condition vs. time-pressure condition, Rand et al. (2012, 2014)). Though a related study reported no connections between prosociality and the disjunction effect (Pothos et al., 2011), the finding is so far controversial. Firstly, the study focused on the prosocial-bias variation across different types of social–economic games, rather than across the information conditions within the Prisoner’s Dilemma that directly give rise to the disjunction effect. Secondly, treating changes in cooperation rates as a proxy for shifts in prosocial bias is problematic, as cooperative behavior reflects the combined influence of both intuitive prosocial tendencies and deliberative processing (Evans, 2008; Bear & Rand, 2016; Rand, 2016). Thirdly, the measurement of prosocial trait in this study was the general personality scale (i.e., agreeableness measured by Self-Description Inventory for Big-5 factors), which might be too coarse to capture the specific prosocial tendencies relevant to strategic cooperation.

To fill this research gap, our study investigates the underlying role of prosociality in the disjunction effect. The effect size of the disjunction effect (EOD) in the PD game can be defined as the average of the defection rates in conditions C and D minus the defection rate in condition U (Pothos et al., 2011; Pothos & Busemeyer, 2009). A larger positive EOD indicates stronger tendency for behavior under uncertainty to deviate toward cooperation. We focus on three key questions: (a) to what extent can the disjunction effect be accounted for by variations in prosocial across conditions; (b) at the aggregate level, whether there is significant alteration of prosocial bias between condition U and two certain conditions, given that information about the opponent’s action can serve as an extrinsic incentives that modulates prosocial bias; (c) at the individual level, whether participants with different levels of prosocial trait show distinct patterns of prosocial-bias variation and defection-rate change, considering that there were discrepancies in the behavioral patterns across prosocial trait profiles (e.g., Mischkowski & Glöckner, 2016)?

It should be noted that measuring prosocial bias is challenging. Some researchers have taken response time (RT) as a reflection of prosocial bias (e.g., Piovesan & Wengström, 2009; Mischkowski & Glöckner, 2016). Such an assumption, however, has been argued against, as there are other influencing factors for RT besides prosocial bias, such as deliberative preference strength (Krajbich et al., 2015). Recently, the drift diffusion model (DDM) shed a light on this problem (Chen & Krajbich, 2018; Gallotti & Grujić, 2019). The DDM assumes that humans make decisions by gradually accumulating noisy information until a threshold of evidence is reached (Ratcliff et al., 2016). By taking the distributions of response pattern and RTs into consideration, the model can disentangle different influencing factors that are essential to the decision process (Forstmann et al., 2016): (i) drift rate (v) represents the average amount of evidence accumulated per unit time; (ii) bias (z) represents a priori inclination for one or the other choice alternative; and (iii) threshold (a) is the quantity that quantifies response caution. In the case of the PD game, drift rate (v) indicates deliberative preference strength for defection vs. cooperation, and bias (z) captures intuitive bias toward self-interest or prosociality. The DDM framework lets us disentangle these components when people make simultaneous decisions in the PD.

We selected social-value orientation (SVO), a relatively stable personality trait (Van Lange, 2000), as the indicator of prosocial trait in the present study. SVO reflects how the individual evaluates interdependent outcomes for oneself and the other, making it highly relevant to decision-making in the PD game. Prior work suggests that individuals with intermediate levels of prosociality experience stronger conflicts between prosocial and selfish motives under uncertainty (Yamagishi et al., 2017), compared with highly prosocial or highly proself individuals. Consequently, these intermediate participants may exhibit larger shifts in prosocial bias when informed of their opponent’s actions, potentially producing a U-shaped or inverted U-shaped relationship between prosocial trait and changes in prosocial bias. Based on this theoretical expectation, we employed quadratic regression to capture the potential nonlinear association between SVO and variations in prosocial bias.

## 2. Methods

### 2.1. Participants

Ninety-one college students with diverse majors were initially recruited, and nineteen of them were then excluded due to (a) abnormal high response consistency and (b) abnormal fast response time. Seventy-two valid participants (33 males and 39 females, M = 21.90, SD = 1.90, range of 19–26 years) participated in the experiment. Participation was by informed consent, and the experimental setup was approved by the Ethics Committee for Scientific Research at the corresponding author’s institution. All methods performed were in accordance with the Declaration of Helsinki. Experimental sessions lasted around 40 min, in which subjects earned on average USD 5.

### 2.2. Prosocial Trait Measurement

The six-item SVO (social value orientation) Slider Measure (Murphy et al., 2011) was adopted for the measurement of SVO. It can produce continuous data and allows for greater explanatory potential through increased statistical power (Murphy & Ackermann, 2014). For each item, participants decided how to allocate a monetary amount between themselves and their anonymous other. Based on participants’ decisions, continuous SVO angles can be calculated, and a higher SVO angle reflects higher prosocial trait (see Murphy et al., 2011 for computation process in detail). For illustration purposes, we need two cutoffs to divide the whole sample into three groups (i.e., proself, intermediate, and prosocial participants). Considering the normality of our sample (Figure S1 in the Supplementary Materials) and that the mean of participants’ SVO angle (20.86°) is close to the middle point the SVO Slider Measure scale (22.45°), which is the boundary for proself and prosocial individuals, we used one standard deviation to set these two cutoffs, meaning that participants with an SVO angle smaller than M − 1SD belong to the proself group (N = 12), those with an SVO larger than M + 1SD belong to the prosocial group (N = 12), and the rest belong to the intermediate group.

### 2.3. Payoff Structure

The prototype payoff matrix (Table S1 in the Supplementary Materials) in our study is similar to that in Shafir and Tversky’s (1992) study. We generated 150 payoff matrices (“payoffs.csv” in Supplementary Materials) by adding a random integer (−10 to +10) to each payoff of the prototype payoff matrix, and then we randomly assigned 50 matrices to each condition (U, D, and C). We ensured that the canonical PD inequalities (Temptation > Reward > Punishment > Sucker; T > R > P > S) were preserved. In practice, we automatically filtered out any matrices that violated these conditions after randomizing values. Across matrices, the mean (±SD) differences were (T − R) = 19.16 ± 8.63, (R − P) = 12.42 ± 7.03, (P − S) = 17.79 ± 7.66, and (T − S) = 49.38 ± 8.21, indicating that the temptation and sucker’s gaps remained positive in all cases.

### 2.4. Experimental Procedure

Prior to the experiment, participants were asked to complete the six-item SVO Slider Measure. To avoid the influence of the SVO measure on the subsequent experiment, there was a more-than-one-week interval between them. In addition, the participants were asked to answer a set of control questions to make sure they understood the game before the formal experiment. The formal experiment was performed on z-Tree, a software for developing and conducting economic experiments (Fischbacher, 2007). The following experiment contains a total of 150 rounds of PD game (50 rounds per condition randomly arranged): U (uncertain, participants received no information about the opponent’s action); D (defector-known, participants were informed that the opponent had chosen to defect); and C (cooperator-known, participants were informed that the opponent had chosen to cooperate). In each round of the game, participants first saw a waiting screen informing them that they were matching a randomly selected, anonymous opponent on the local area network (which in fact did not exist) until they were informed with successful match after a period of time (3~10 s in our setting). Participants were required to press “F” on the keyboard if they selected defection and press “J” if they selected cooperation option. Then, they were required to press “Spacebar” to move to the next trial. After that, they saw the waiting screen for the next round. At the end of the experiment, participants were shown a screen informing them of their total earnings of the 150 rounds (see screenshots presented in the Supplementary Materials for detailed experimental procedure).

### 2.5. Drift Diffusion Model

**Figure 1:**
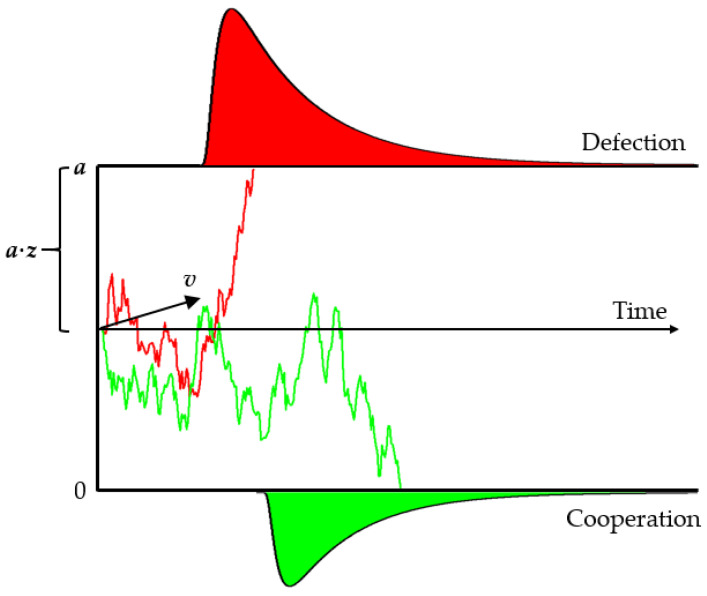
Graphical illustration of the drift diffusion model. The decision process starts at a·(1 − z) and accumulates with drift v (red: drift toward defection boundary a, green: toward cooperation at 0). Thick curves show the RT distributions for defection (red) and cooperation (green). High v means evidence strongly favors defection; high z (starting closer to a) means an initial bias to defect (selfishness).

We fit the drift diffusion model (DDM) to participants’ choices and response times. The DDM assumes that an evidence variable, X(t), accumulates until a boundary is reached (Ratcliff et al., 2016, see Figure 1 for graphical illustration). The evidence, X(t), follows a diffusion process and evolves in small time increments according to a stochastic difference equation, X(t + 1) = X(t) + v + W(t) (with discrete time this is technically a random walk model), where v is the drift rate (mean evidence per unit time favoring defection vs. cooperation), and W(t) is mean-zero Gaussian noise. The process starts at X(0) = z × a (0 ≤ z ≤ 1, with a the boundary separation) and stops when X(t) first hits 0 (cooperate decision) or a (defect decision). Thus, the drift rate (v) captures the deliberative preference for defection (higher v indicates stronger push to defect), while the starting point (z) captures initial bias: z = 0.5 is neutral, z > 0.5 means a bias toward defection (selfish), and z < 0.5 means a bias toward cooperation (prosocial). The boundary separation reflects caution (bigger a indicates slower, more cautious decisions). In our PD context, we interpret z as the participant’s intuitive prosocial/selfish bias according to Chen and Krajbich (2018). We expect to see z shift depending on knowledge of the opponent’s move, revealing intuitive bias changes. 

We estimated these DDM parameters for each subject and condition using the DstarM package, which implements the D∗M method (Verdonck & Tuerlinckx, 2016). In this approach, the model predictions are summarized by a quantile-probability function (QP); for example, the 0.1, 0.3, 0.5, 0.7, and 0.9 RT quantiles are computed for each response type (cooperation vs. defection). The fitting algorithm minimizes a goodness-of-fit statistic (specifically, a chi-square distance) between the observed and model-predicted RT distributions. A global Differential Evolution optimizer is used to find the best-fitting v, a, and z (and a common T_er_ across conditions) while matching these quantile-probability functions. This procedure effectively aligns the shape of the model’s RT distributions with the empirical data. We checked model fit by plotting the empirical vs. predicted RT quantiles (as recommended by Van den Bergh et al., 2020), and we found that the DDM captured the data well in all condition–response combinations (Figure S2 in the Supplementary Materials).

### 2.6. Statistical Analysis

For analysis in the aggregate level, the one-way repeated ANOVA^1^ and the Bonferroni post hoc test were used to test the aggregate difference in defection rates, thresholds, drift rates, and bias among different conditions. Partial eta-squared ($\eta_{P}^{2}$) was reported as a measure of effect size of the ANOVA. Then, multiple regression of the relationship between defection-rate variation and its corresponding threshold, drift rate, and bias variations was adopted to derive how much of the defection-rate variation can be explained by the variations in the corresponding DDM parameters. The relative importance metric PMVD, referring to the quantification of an individual regressor’s contribution to the whole regression model (Grömping, 2007), was used to quantify such contribution of different DDM parameter variations to the regression with defection-rate variation. For the analysis at the individual level, we first adopted the correlation analysis to identify the relationships between SVO and (i) derived DDM parameters and (ii) defection rates. Then, both linear and quadratic regressions were adopted to explore the relationships between SVO and variations in prosocial bias, as well as the defection rates. Furthermore, the mediation analysis was performed to test the mediation role of prosocial-bias variation in the relationship between SVO and defection-rate variation. All statistical analysis were conducted in R, with the additional requirement of rstatix (https://cran.r-project.org/web/packages/rstatix, access data: 18 October 2025), psych (https://cran.r-project.org/web/packages/psych, access date: 23 June 2025), relaimpo (https://prof.bht-berlin.de/groemping/software/relaimpo/, access date: 4 October 2023), and mediation packages (https://cran.r-project.org/web/packages/mediation, access date: 7 June 2025).

## 3. Results

### 3.1. Test of the Disjunction Effect

The means and distributions of the defection rates under different conditions are displayed in Figure S2 in the Supplementary Materials. The averaged defection rates for conditions D, C, and U are 80.1%, 69.9%, and 64.1%, respectively. The one-way repeated-measures ANOVA with GG correction showed a significant effect of condition type for defection rates [F(1.64, 116.16) = 79.896, *p* < 0.001, $\eta_{P}^{2}$ = 0.529]. The following Bonferroni post hoc test revealed significant differences of defection rates between condition D and C (t = −10.875, df = 71, *p* < 0.001), between condition D and U (t = −10.997, df = 71, *p* < 0.001), and between condition C and U (t = −4.193, df = 71, *p* < 0.001). These results demonstrate a robust disjunction effect in our study.

### 3.2. DDM Analysis

#### 3.2.1. Threshold

The one-way repeated ANOVA showed no significant effect of condition type on thresholds, F(2, 142) = 0.920, *p* = 0.401, and $\eta_{P}^{2}$ = 0.013, indicating there is no significant alteration of response caution across different conditions.

#### 3.2.2. Drift Rate

**Figure 2:**
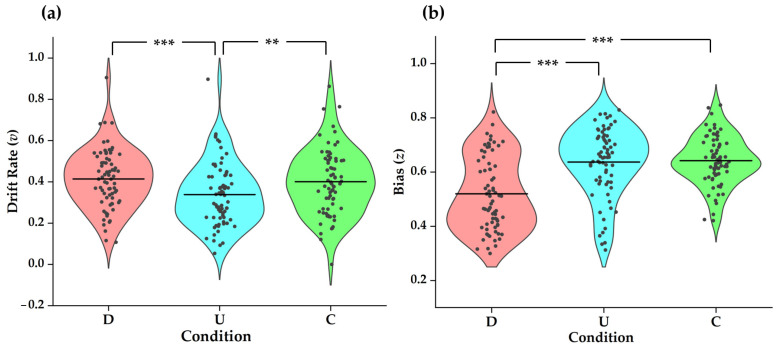
Drift rate and bias under different conditions. (**a**) Drift rate, v, was highest in the known conditions (D and C) and lower in uncertainty (U), indicating a reduced defection bias under uncertainty. (**b**) Bias, z, was lowest in D, higher in C and U, reflecting reduced prosocial bias when knowing the opponent defects. Note: ** and *** represent *p* < 0.01 and *p* < 0.001, respectively.

The one-way repeated ANOVA showed a significant effect of condition type on drift rate, F(2, 142) = 8.535, *p* < 0.001, and $\eta_{P}^{2}$ = 0.107. The following Bonferroni post hoc test revealed significant alterations of drift rate between condition D and U (t = 3.891, df = 71, *p* < 0.001) and between condition C and U (t = 3.371, df = 71, *p* = 0.004), as displayed in Figure 2a. The results indicate a significant reduction of deliberate preference toward the defective choice under condition U.

#### 3.2.3. Bias

The one-way repeated ANOVA on the bias showed a significant effect of condition type [F(2, 142) = 71.809, *p* < 0.001, $\eta_{P}^{2}$ = 0.503]. The following Bonferroni post hoc test showed significant differences of bias between condition D and C (t = −9.940, df = 71, *p* < 0.001) and between condition D and U (t = −11.192, df = 71, *p* < 0.001), as displayed in Figure 2b, indicating a significant increase in prosocial bias in conditions C and U compared with condition D. To address the bounded nature of bias, we also conducted the ANOVA on the logit-transformed bias. The one-way repeated ANOVA on transformed z showed equivalent significance [F(2, 142) = 69.616, *p* < 0.001, $\eta_{P}^{2}$ = 0.495] and pattern (D–C: t = −9.870, df = 71, *p* < 0.001; D–U: t = −11.285, df = 71, *p* < 0.001; U–C: t = −0.219, df = 71, *p* = n.s.), confirming that our results are robust to this transform.

### 3.3. The Contribution of Each DDM Parameter Variation to EOD

The multiple regression statistics of the relationship between the defection-rate variations and the DDM parameters variations, as well as the contribution metric PMVD for each regressor, are displayed in Table 1. We observed that the defection-rate variations were significantly positively associated with the corresponding drift-rate variations and significantly negatively associated with the corresponding bias variations. Moreover, we found that majority variance of the defection-rate variations (80.9~90.3%) can be explained by the corresponding DDM parameter variations, in which the drift-rate variations made a relatively larger contribution (60.2~65.3%), and the bias variations made a relatively smaller but substantial contribution (33.1~33.9%). These findings indicated that both preference strength toward defection variation and prosocial-bias variation played essential roles in the production of the disjunction effect.

### 3.4. The Relationship Between SVO and (a) DDM Parameters and (b) Defection Rates

The correlation analysis showed that (i) there were significant linear associations between SVO and prosocial bias ($z_{d}$: r = 0.437, *p* < 0.001; $z_{c}$: r = 0.246, *p* = 0.037; $z_{u}$: r = 0.517, *p* < 0.001), suggesting the individuals with higher prosocial trait are of higher prosocial bias regardless of the conditions; (ii) there was no significant linear association between SVO and thresholds, or between SVO and drift rates, indicating the independence between SVO and these DDM variables; and (iii) significant negative linear correlations were found between SVO and defection rates (p(Defect|D): r = −0.279, *p* = 0.017; p(Defect|C): r = −0.281, *p* = 0.016; p(Defect|U): r = −0.360, *p* = 0.002), indicating individuals with higher prosocial trait tend to cooperate more regardless of the conditions.

### 3.5. The Relationship Between SVO and Prosocial-Bias Variations

**Figure 3:**
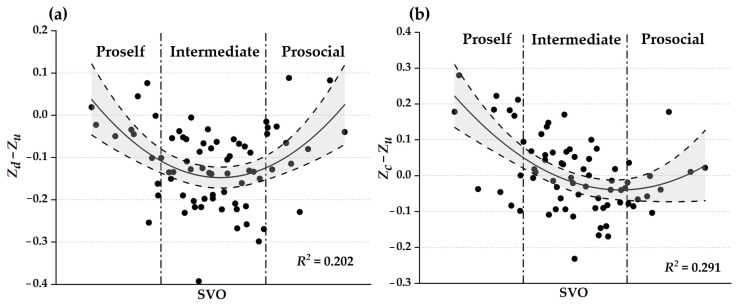
The quadratic relationships between SVO and prosocial-bias variation: (**a**) bias variation from U to D and (**b**) from U to C. Gray area representing 95% confidence interval (CI). Intermediate SVO shows the largest negative Δz, yielding an inverted-U relationship.

The results of both linear and quadratic regression analysis for the relationships between SVO and prosocial-bias variations are presented in Table 2, and they favor the quadratic U-shaped relationships instead of linear relationships between SVO and the prosocial-bias variation between condition D and U ($z_{d}-z_{u}$), between SVO and the prosocial-bias variation, and between condition C and U ($z_{c}-z_{u}$), because of the significance of the coefficients of quadratic terms and higher adjusted R-squared in the quadratic regression. The graphical illustrations of these quadratic relationships are displayed in Figure 3. These results showed, taking condition U as baseline, proself participants showed a marginal decrement of prosocial bias when knowing the opponent’s action is defection (M = −0.060, SD = 0.099, t = −2.101, *p* = 0.060, paired sample t-test) and moderate increment of prosocial bias when knowing the opponent’s action is cooperation (M = 0.097, SD = 0.137, t = 2.443, *p* = 0.033); intermediate participants showed substantial decrement of prosocial bias under condition D (M = −0.147, SD = 0.080, t = −12.789, *p* < 0.001) and little decrement under condition C (M = −0.012, SD = 0.089, t = −0.943, *p* = 0.350); and prosocial participants showed marginal decrement of prosocial bias under condition D (M = −0.050, SD = 0.087, t = −2.013, *p* = 0.069) and little decrement of prosocial bias under condition C (M = −0.017, SD = 0.076, t = −0.779, *p* = 0.453).

### 3.6. The Relationship Between SVO and EOD Through Prosocial-Bias Variations

**Figure 4:**
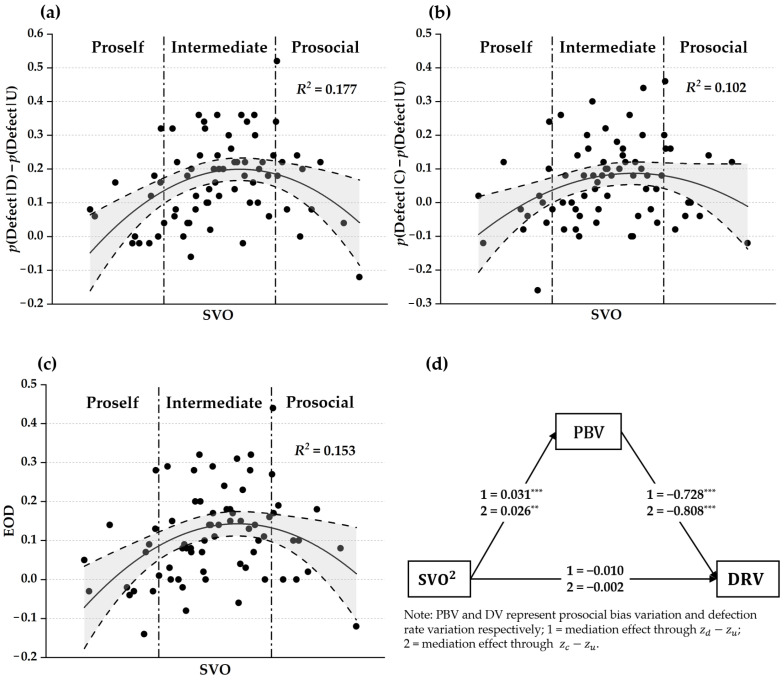
The quadratic relationships between SVO and (**a**) p(Defect|D)−p(Defect|U), (**b**) p(Defect|C)−p(Defect|U), and (**c**) EOD. (**d**) The mediation effects of prosocial-bias variation (PBV) on the quadratic relationship between SVO and defection-rate variation (DRV). Note: ** *p* < 0.01, *** *p* < 0.001.

As there is a strong negative linear linkage between prosocial-bias variations and defection-rate variations (the negative associations displayed in Table 1) and the independence between SVO and other DDM variables, we hypothesize the quadratic relationships between SVO and prosocial-bias variations might lead to similar quadratic relationships between SVO and defection-rate variations (i.e., p(Defect|D)−p(Defect|U) and p(Defect|C)−p(Defect|U)), which will eventually result in a quadratic relationship between SVO and EOD. The regression results presented in Table 3 demonstrate such inverted U-shaped relationships. The graphical illustrations of these quadratic inverted U-shaped relationships are displayed in Figure 4a–c, showing that when taking condition U as the baseline, proself participants defected marginally more (M = 0.085, SD = 0.107, t = 2.763, *p* = 0.019) under condition D and cooperated little more under condition C (M = −0.007, SD = 0.127, t = −0.172, *p* = 0.857), thus resulting in a small EOD (M = 0.039, SD = 0.111, t = 1.219, *p* = 0.247); and intermediate participants defected substantially more under condition D (M = 0.178, SD = 0.111, t = 11.162, *p* < 0.001) and condition C (M = 0.072, SD = 0.107, t = 4.616, *p* < 0.001), resulting in a large EOD (M = 0.125, SD = 0.102, t = 8.382, *p* < 0.001); prosocial participants defected moderately more under condition D (M = 0.167, SD = 0.167, t = 3.447, *p* = 0.006) and defected a little bit more under condition C (M = 0.072, SD = 0.141, t = 1.767, *p* = 0.105), resulting in a medium EOD (M = 0.119, SD = 0.146, t = 2.826, *p* = 0.017).

We then performed mediation analysis to compute how much of the quadratic relationships between SVO and defection-rate variations can be explained by the variations in the prosocial bias. The results of the mediation effects of both $z_{d}-z_{u}$ and $z_{c}-z_{u}$ on the quadratic relationships between SVO and corresponding defection-rate variations are displayed in Figure 4d and Table 4, demonstrating a strong mediating role of prosocial-bias variations on the quadratic relationships between SVO and defection-rate variations.

## 4. Discussion

By adopting the DDM model, our study revealed that the disjunction effect can be well explained by the variations in several DDM parameters (i.e., preference strength and prosocial bias), suggesting that prosocial-bias variation is also a valid cause of the disjunction effect and thus supporting the dual-process research highlighting the involvement of both deliberation and intuition in social economic decision-making (e.g., Rand et al., 2012; Fiedler et al., 2013; Lohse et al., 2017). The excellent explanation rate of the disjunction effect from preference strength and prosocial-bias variations is consistent with the advantage of the DDM framework that the parameters derived from the DDM model are mathematically robust and precise (Chen & Krajbich, 2018).

At the aggregate level, we found significant alterations of drift rates and prosocial bias across conditions. For the drift rate, we observed a significant reduction in condition U, which is consistent with previous studies suggesting that participants’ preference strength toward defection choice somehow reduces under condition U (Shafir & Tversky, 1992; Croson, 1999; Li & Taplin, 2002; Li et al., 2007; Hristova & Grinberg, 2010). Importantly, we observed a significant reduction of prosocial bias from condition U to condition D, and an insignificant alteration from condition U to condition C. This asymmetry suggests that participants became more selfish when they knew their opponents had chosen the selfish option, while they did not become more prosocial when they knew their opponents had chosen the prosocial option. This finding is also consistent with that of the intermediate participants because they occupy the largest proportion (66.7%) of our sample, and the reason for the production of such variation pattern will be discussed in the following section.

At the individual level, we observed that intermediate-SVO participants had the largest bias shifts (especially U → D) compared to high- or low-SVO individuals. This in turn produced an inverted-U relationship between SVO and defection rate changes (and effect size), as intermediates were most influenced by knowing the opponent defected. Mediation analysis confirmed that the quadratic relation between SVO and bias change accounted for most of the relationship between SVO and cooperation changes. In other words, the heterogeneity in bias change largely underlies the heterogeneity in defection behavior and hence the disjunction effect across individuals. This supports the idea that cognitive dissonance between “wishful” expectations and feedback drives the bias shift: participants expected opponents similar to themselves (wishful thinking), so surprising defection led to large bias adjustments. Prosocial individuals showed the smallest bias shifts, consistent with prior findings that cooperators are less perturbed by others’ actions.

We proposed the following intuitive expectation—feedback-information conflict theory for the possible explanation for individual differences in prosocial-bias variations. In social interactions, individuals intuitively expected that their opponents would make decisions based on the similar prosocial bias as themselves in the regular social economic games (i.e., the feedback information about their opponents’ actions are not clear) (Kelley & Stahelski, 1970; Declerck & Bogaert, 2008; Qi et al., 2017), meaning that proself, intermediate, and prosocial participants tend to expect their opponents to choose the selfish (defection), reciprocal (cooperation), and altruistic (cooperation) option, respectively, under condition U. Shafir and Tversky (1992) referred to such prosocial bias-based intuitive expectation as “wishful thinking” in their original work on the disjunction effect. When the feedback information about an opponent’s action is consistent with a participant’s expectation, that participant need not change his/her predisposed prosocial bias; thus, there was no significant prosocial-bias alteration between condition U and the corresponding conditions in which opponents’ actions are consistent with participants’ expectations. When the feedback information is inconsistent with participants’ expectation, such a conflict might result in cognitive dissonance (Festinger, 1957). To resolve such cognitive dissonance, the participant needs to change his/her prosocial bias to be more consistent with the feedback information. Therefore, we observed larger prosocial-bias variations under such inconsistency compared with those under consistency. It should be noted that there are also individual differences in such prosocial-bias variations: the largest alteration of prosocial bias is from intermediate participants, indicating they are more sensitive to intuitive expectation–feedback information conflict compared with participants with extreme SVO. We also observed the smallest alteration from prosocial participants, a finding that is consistent with the previous studies showing that prosocials are less sensitive to feedback information compared with proself individuals when making decisions in social economic games (Boone et al., 2010; Emonds et al., 2011; Declerck et al., 2013).

Importantly, our findings fit into the broader literature on prosociality. Capraro (2013) proposed that humans have an innate cooperative predisposition, so they consider coalition payoffs rather than acting purely as lone agents. This predicts nonzero cooperation even in one-shot games, consistent with our finding of a baseline prosocial bias in U. Capraro (2015) further found that about one-sixth of people exhibit hyper-altruism, preferring to give rather than take even when it is costly. Such strong prosocial tendencies support the idea that intuitive bias toward helping is common. Recent evolutionary models provide a possible rationale: Salahshour and Couzin (2025) showed that altruistic rationality can evolve if agents distort their payoffs subjectively, leading them to perceive social dilemmas as coordination games. Similarly, Salahshour (2025) describes a perceptual rationality framework in which rational decisions are based on evolvable perceptions of social environment. These theories imply that prosocial inclinations could be adaptive and coherent with rationality—aligning with our view that prosocial bias is not mere error. Additionally, strong reciprocity theory posits that humans are predisposed to cooperate and to punish defectors even when it is not personally beneficial. This inherent inclination may underlie the prosocial starting-point bias we estimate. Finally, one recent model of the disjunction effect (i.e., Markov belief model in Xin et al., 2022) and our sequential-sampling perspective both highlight the role of intuitive bias, especially the altered bias in U in explaining the disjunction effect. Overall, our results suggest that intuitive prosocial biases are deep-rooted and may even be considered rational under certain subjective frameworks.

There are several limitations of our study to be addressed. Firstly, our interpretation of z as prosocial bias could be confounded by other factors. For example, the fixed response mapping (press “F” = defect [left key]; “J” = cooperate [right key]) might induce a motor or spatial bias at the starting point. Sequential effects (e.g., a tendency to repeat previous choices) can also shift the starting point on each trial, so part of the z differences might reflect carryover effects. In future work, randomizing response keys or modeling trial history will help isolate pure prosocial bias. Secondly, our study fails to manipulate the degree of uncertainty (this is also the limitation for the most of relevant studies at present); therefore, a detailed and quantitative description about how prosocial bias and EOD vary across different levels of uncertainty cannot be derived. Future studies may explore this issue by improving the uncertainty representation in the current experimental framework. Thirdly, considering cooperation in various interaction contexts can be highly culturally variable (Henrich et al., 2005), and the response to uncertainty can also vary between cultures (Lau & Ranyard, 2005; Weber & Hsee, 2000), so it is possible that the patterns we have found are not universal because of the cultural homogeneity of our sample. Future studies using culturally different samples can be conducted to examine this issue. Fourthly, we did not adopt other nonlinear specifications (e.g., spline and exponential models) to explore the inverted U-shaped relationship, so future work may investigate whether more flexible functional forms yield stronger or more interpretable effects. Another limitation is that we fit each participant’s data separately using the D* M method (via the DstarM package). This approach has certain advantages (Van den Bergh et al., 2020)—for example, it “circumvents specifying a distribution for the nondecision processes” by leaving the nondecision time distribution unspecified, and it uses a global optimization on the full RT distributions. Van den Bergh et al. (2020) showed in simulations that this method can accurately recover diffusion parameters (and nondecision times) under these relaxed assumptions. However, because each subject is fit independently, DstarM forfeits the benefits of hierarchical pooling. Hierarchical Bayesian models (such as HDDM) constrain individual parameters by group-level distributions, and doing so typically stabilizes estimates when data are sparse (Wiecki et al., 2013). A prior study also found that hierarchical fits require far fewer trials per subject and yield more accurate parameter recovery than non-hierarchical methods (Jin et al., 2025). We chose DstarM fitting to retain subject-specific estimates, and because our sample and effect sizes are moderate. Nevertheless, future HDDM analysis could test the robustness of our findings under a hierarchical framework.

In conclusion, we have shown that prosocial biases, as revealed by the DDM starting point, play a significant role in the disjunction effect. This highlights that humans’ intuitive leaning toward or against cooperation—possibly rooted in evolved social preferences—can sway decisions beyond pure payoff reasoning. Our work clarifies how prosociality and decision dynamics interact, and connects psychological modeling to economic and evolutionary theories of cooperation

## Figures and Tables

**Table 1:** Regression statistics for the relationships between defection-rate variations and their corresponding DDM parameter variations. Note: *** *p* < 0.001 hereinafter.

Outcome	$p\left(Defect|D\right)-p\left(Defect|U\right)$		Outcome	$p\left(Defect|C\right)-p\left(Defect|U\right)$	
Predictor	β	PMVD	Predictor	β	PMVD
$a_{d}-a_{u}$	0.018	1.6%	$a_{c}-a_{u}$	0.039	5.9%
$v_{d}-v_{u}$	0.634 ***	65.3%	$v_{c}-v_{u}$	0.622 ***	60.2%
$z_{d}-z_{u}$	−0.809 ***	33.1%	$z_{c}-z_{u}$	−0.797 ***	33.9%
*R* ^2^	0.809		*R* ^2^	0.903	
*F* statistic	92.556 ***		*F* statistic	213.257 ***	

**Table 2:** Linear and quadratic regression statistics of the relationships between SVO and prosocial-bias variation. Note: ** *p* < 0.01, *** *p* < 0.001 hereinafter.

$\mathrm{The}\;\mathrm{Relationship}\;\mathrm{Between}\;\mathrm{SVO}\;\mathrm{and}\;z_{d}-z_{u}$				
**Predictors**	**Linear Model**		**Quadratic Model**	
	β	**95% CI**	β	**95% CI**
Intercept	−0.117 ***	−0.139, −0.095	−0.147 ***	−0.172, −0.122
SVO	−0.007	−0.029, 0.015	−0.008	−0.028, 0.012,
SVO^2^			0.031 ***	0.016, 0.046,
Adjusted *R*^2^	−0.009		0.179	
*F* statistic	0.383		8.732 ***	
**The Relationship Between SVO and** $z_{u}-z_{c}$				
**Predictors**	**Linear Model**		**Quadratic Model**	
	β	**95% CI**	β	**95% CI**
Intercept	0.005	−0.017, 0.027,	−0.020	−0.046, 0.005
SVO	−0.043 ***	−0.066, −0.021	−0.043 ***	−0.066, −0.023
SVO^2^			0.026 **	0.010, 0.041
Adjusted *R*^2^	0.167		0.270	
*F* statistic	14.13 ***		15.17 ***	

**Table 3:** Linear and quadratic regression statistics for the relationships between SVO (normalized) and the variations in cooperation rates, as well as EOD. Note: * *p* < 0.05, ** *p* < 0.01, *** *p* < 0.001 hereinafter.

The Relationship Between SVO and p(Defect|D)−p(Defect|U)				
**Predictors**	**Linear Model**		**Quadratic Model**	
	β	**95% CI**	β	**95% CI**
Intercept	0.161 ***	0.132, 0.190	0.195 ***	0.162, 0.227
SVO	0.023	−0.006, 0.052	0.023	−0.002, 0.052
SVO^2^			−0.034 ***	−0.053, −0.015
Adjusted *R*^2^	0.022		0.153	
F statistic	2.592		7.441 **	
**The Relationship Between SVO and** p(Defect|C)−p(Defect|U)				
**Predictors**	**Linear Model**		**Quadratic Model**	
	β	**95% CI**	β	**95% CI**
Intercept	0.059 ***	0.031, 0.086	0.082 ***	−0.006, 0.005
SVO	0.021	−0.007, 0.047	0.021	−0.005, 0.047
SVO^2^			0.023 *	−0.043, −0.004
Adjusted *R*^2^	0.016		0.076	
*F* statistic	2.18		3.935 *	
**The Relationship Between SVO and EOD**				
**Predictors**	**Linear Model**		**Quadratic Model**	
	β	**95% CI**	β	**95% CI**
Intercept	0.110 ***	0.083, 0.136	0.138 ***	0.107, 0.169
SVO	0.022	−0.005, 0.047	0.023	−0.002, 0.047
SVO^2^			−0.029 **	−0.047, −0.010
Adjusted *R*^2^	0.023		0.130	
*F* statistic	2.673		6.298 **	

**Table 4:** Direct, indirect, and total effects of quadratic SVO term on defection-rate variations. Note: ** *p* < 0.01, *** *p* < 0.001 hereinafter.

Outcome Variable	p(Defect|D)−p(Defect|U)		p(Defect|C)−p(Defect|U)	
	β	95% CI	β	95% CI
Direct effect	−0.010	−0.019, 0.000	−0.002	−0.007, 0.007
Indirect effect	−0.022 ***	−0.039, −0.013	−0.021 **	−0.036, −0.007
Total effect	−0.033 ***	−0.047, −0.020	−0.023 **	−0.035, −0.008
Proportion of indirect effect	68.2%		89.9%	

Note: 95% CI represents 95% confidential interval calculated by the bias-corrected and accelerated (BCa) bootstrapping method.

## Data Availability

The original contributions presented in this study are included in the article and Supplementary Materials. Further inquiries can be directed to the corresponding author.

## Note

1 The Greenhouse–Geisser (GG) correction is adopted if the assumption of sphericity is violated.

## Supplementary Materials

The following supporting information can be downloaded at https://www.mdpi.com/article/10.3390/bs16010132/s1, Figure S1. The distribution of SVO angles (Mean = 20.86°, Kurtosis = −0.21, Skewness = 0.03). Figure S2. Empirical and model predicted quantiles and accuracy for different condition-response combinations. The dot-line represents the quantiles predicted by the DDM model and the markers represents those quantiles calculated from the observed data. Figure S3. The defection rates under different conditions. Note: * *p* < 0.05, ** *p* < 0.01, *** *p* < 0.001, and the lines in the middle of each distribution represent the means. Table S1 The prototype payoff matrix in our study.
