# Supplementary material for: Accounting the Role of Prosociality in the Disjunction Effect with a Drift Diffusion Model
Source: Behav Sci (Basel). 2026 Jan 16;16(1):132. doi: 10.3390/bs16010132 (PMC12838203; doi:10.3390/bs16010132)
Supplement: Supplementary file 1 [file behavsci-16-00132-s001.zip › Supplmentary Material.pdf]

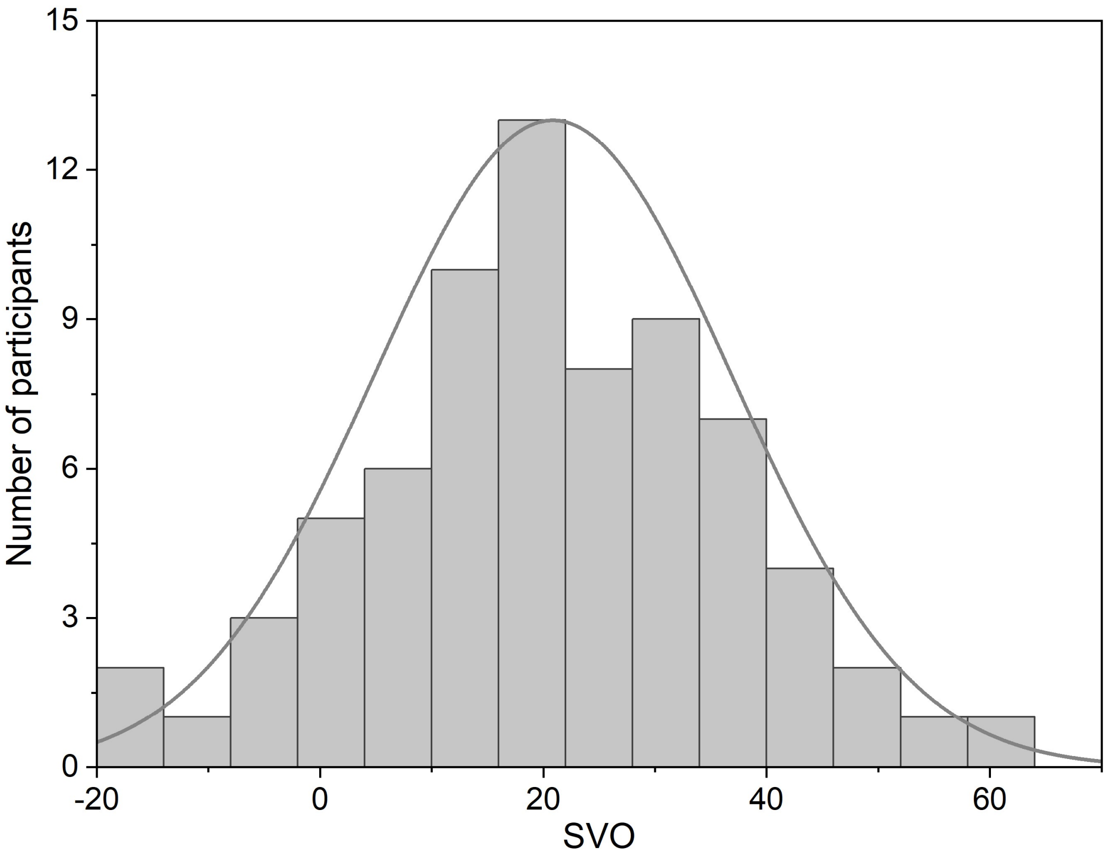

Figure S1. The distribution of SVO angles (Mean = 20.86°, Kurtosis = -0.21, Skewness = 0.03).

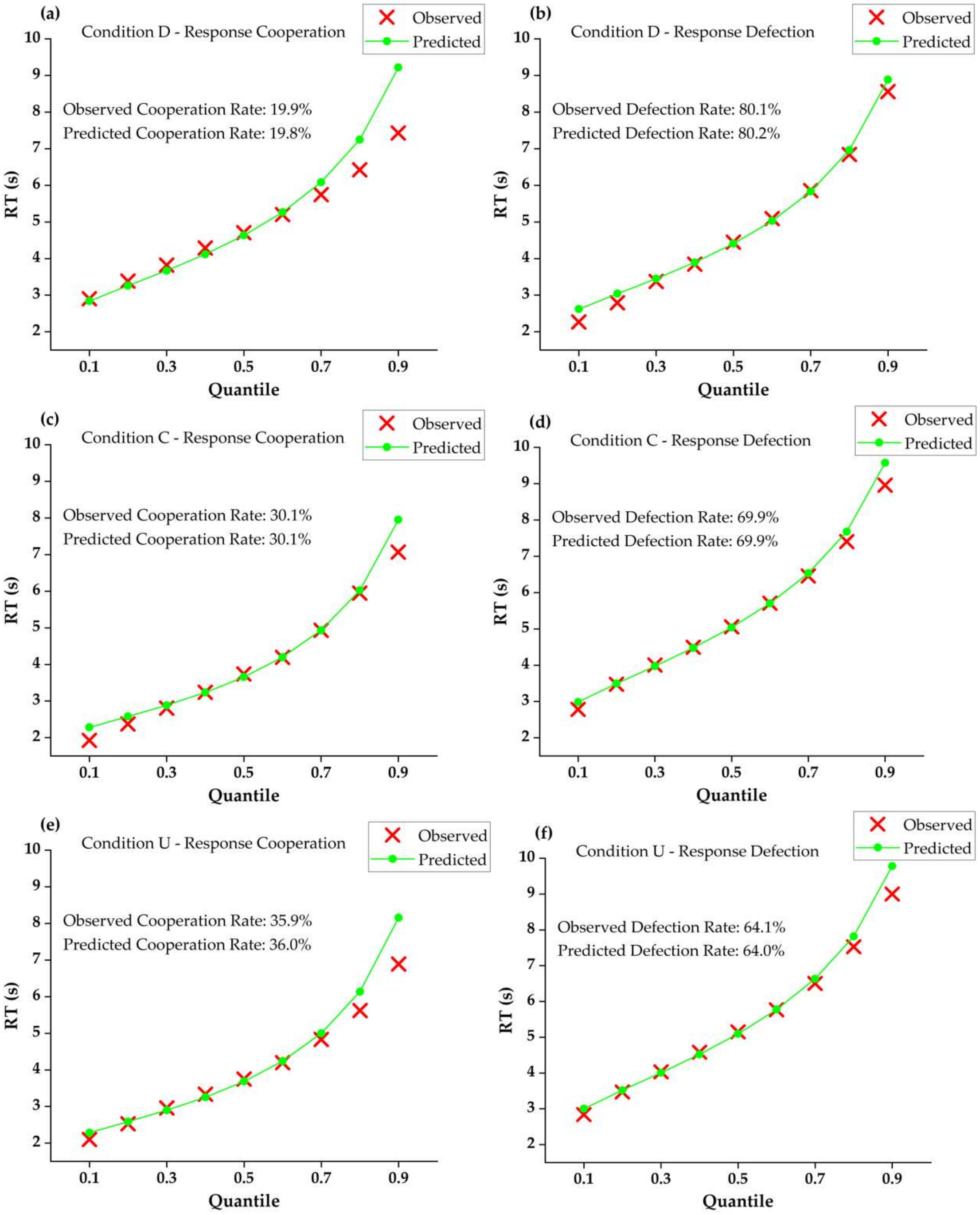

Figure S2. Empirical and model predicted quantiles and accuracy for different condition-response combinations. The dot-line represents the quantiles predicted by the DDM model and the markers represents those quantiles calculated from the observed data.

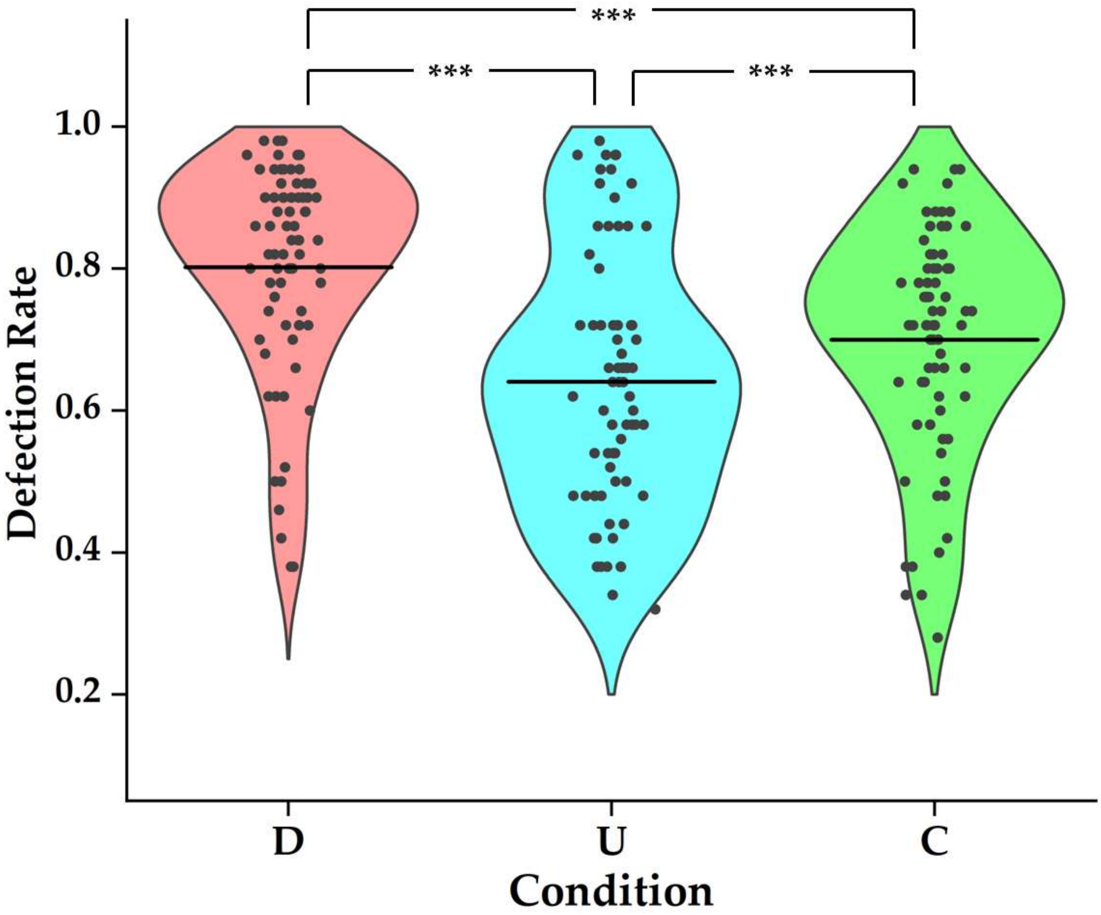

Figure S3. The defection rates under different conditions. Note: * $p < 0.05$, ** $p < 0.01$, *** $p < 0.001$, and the lines in the middle of each distribution represent the means.

Table S1. The prototype payoff matrix in our study, which is set up in the way that no matter what move your opponent makes, you are better off to defect; the same is true for your opponent.

	You defect	You cooperate
The opponent defects	You: 45<br>Opponent: 45	You: 25<br>Opponent: 75
The opponent cooperates	You: 75<br>Opponent: 25	You: 55<br>Opponent: 55

The detailed experimental introduction for participants (language converted from Chinese to English)

Welcome!

Welcome to the online game platform.
This game will last for approximately 40 minutes. During the game it is not allowed to talk with the other participants.
You will earn money in this study. How much you earn depends on your decisions and the decisions of others.
At the end of the game, you will get instructions on how to obtain your payment. We will not inform any of the other participants about your earnings.
If you have a question, raise your hand and we will come to answer it.

Next Page

You will have multiple rounds of game, each matching with a different interaction player from local area Network.
The game is anonymous: you can't find out the real identity of other players, and they can't find this out about you.
In each Round, you must decide whether you will defect or cooperate with the other player. The other player will have to make a similar decision.
Your earning is dependent on not only the decisions made by you and the other player but also the payoff matrix.
One such matrix looks like the following.

	You Defects: F	You Cooperates: J
Other Defects	You: 45<br>Other: 45	You: 25<br>Other: 75
Other Cooperates	You: 75<br>Other: 25	You: 55<br>Other: 55

According to this matrix, if you both cooperate you will both earn 55 points each. If you cooperate and the other player defects, the other player will earn 75 points and you will earn 25 points.
Similarly, if you defect and the other cooperates, you will earn 75 points and the other player will earn 25 points.
Finally, if you both choose to defect, you will earn 45 points each.
To choose defection you would press 'F' key and to choose cooperation you would press 'J' key.

Prevous Page

Next Page

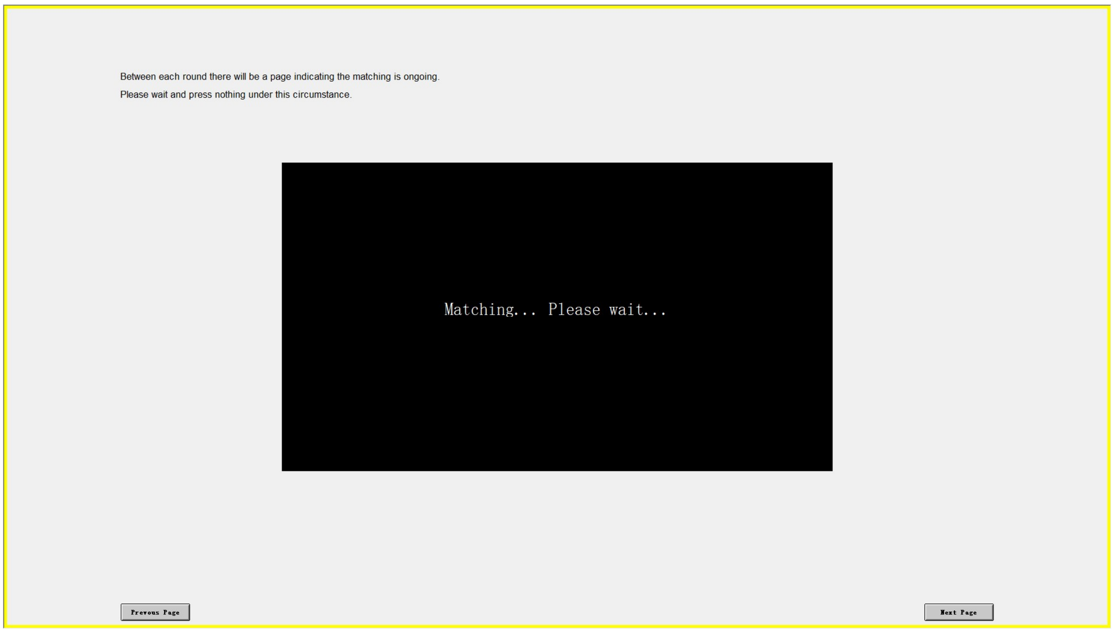
Between each round there will be a page indicating the matching is ongoing.
Please wait and press nothing under this circumstance.
Matching... Please wait...

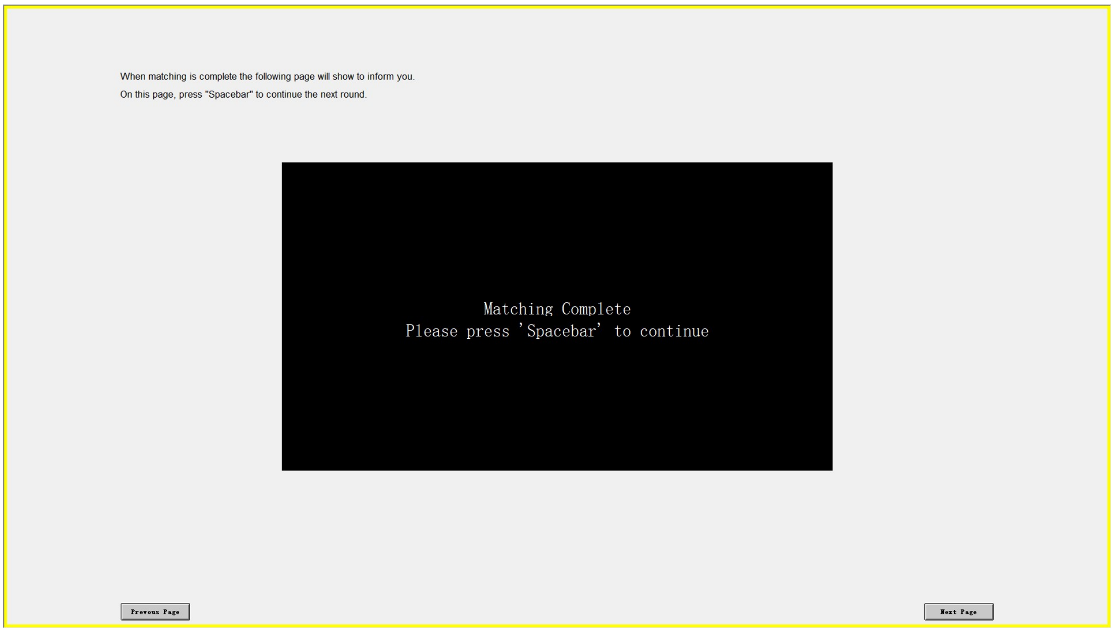
When matching is complete the following page will show to inform you.
On this page, press "Spacebar" to continue the next round.
Matching Complete
Please press 'Spacebar' to continue

Sometimes you will be informed of the strategy that your interaction player has already chosen.
The page below is an example informing that the other player has chosen to defect.
You are free to use the information to help you choose your own strategy.

The other player has chosen to Defect.

	You Defects: F	You Cooperates: J
Other Defects	You: 45<br>Other: 45	You: 25<br>Other: 75
Other Cooperates	You: 75<br>Other: 25	You: 55<br>Other: 55

Prevous Page	Next Page

After every 15 rounds, you will see how many points you earned in those rounds.
At the end of the game, the points that you accumulate will be converted to actual money that will be paid to you at the following rate:

2 points = 1 cent RMB

Before the real experiment starts, a test trial will be presented to make you familiar with the decision making environment on the computer screen.
Please answer the questions presented in this trail. Only if the questions are correctly answered, the experiment will start.

Prevous Page	Next Page

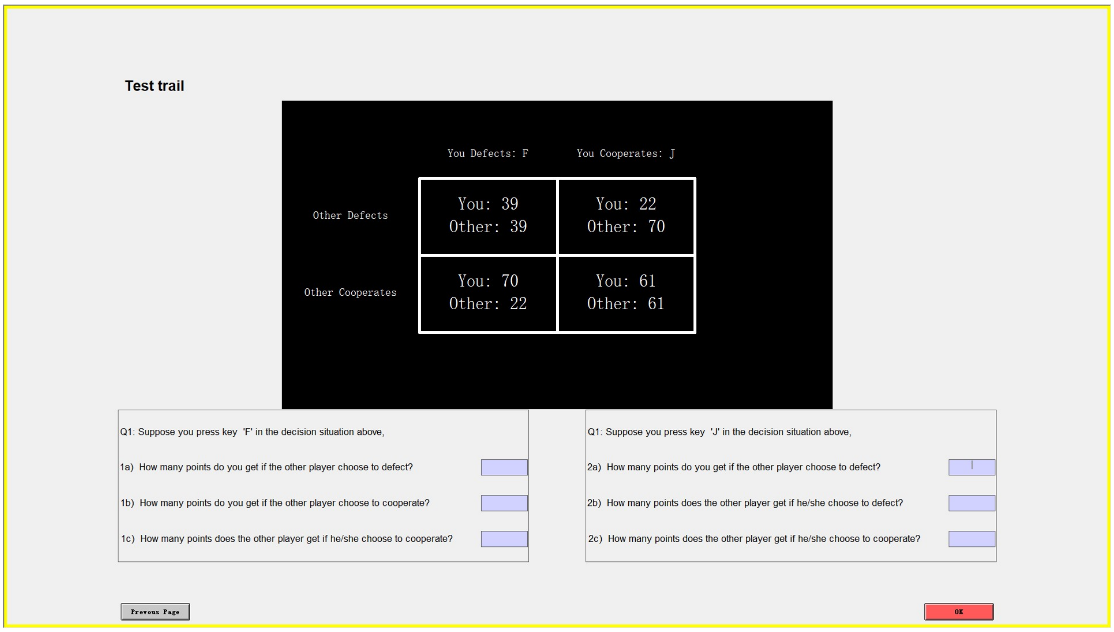
Test trail
You Defects: F
You Cooperates: J
Other Defects
Other Cooperates
You: 39
Other: 39
You: 22
Other: 70
You: 70
Other: 22
You: 61
Other: 61
Q1: Suppose you press key 'F' in the decision situation above,
1a) How many points do you get if the other player choose to defect?
1b) How many points do you get if the other player choose to cooperate?
1c) How many points does the other player get if he/she choose to cooperate?
Q1: Suppose you press key 'J' in the decision situation above,
2a) How many points do you get if the other player choose to defect?
2b) How many points does the other player get if he/she choose to defect?
2c) How many points does the other player get if he/she choose to cooperate?
Prevous Page
OK
